# An Improved Parameter Identification Algorithm for the Friction Model of Electro-Hydraulic Servo Systems

**DOI:** 10.3390/s23042076

**Published:** 2023-02-12

**Authors:** Jian Liao, Fuming Zhou, Jianbo Zheng

**Affiliations:** 1 Institute of Vibration and Noise, Naval University of Engineering, Wuhan 430033, China; 2 Naval Key Laboratory of Ship Vibration and Noise, Naval University of Engineering, Wuhan 430033, China

**Keywords:** improved adaptive genetic identification algorithm, electro-hydraulic servo system, LuGre friction model, parameter identification

## Abstract

Friction is an inherent nonlinear disturbance that can lead to creeping, jitter, and decreased tracking precision in an electro-hydraulic servo system. In this paper, the LuGre friction model is used to describe the dynamic and static characteristics of the friction force of a servo system comprehensively. Accurate identification of model parameters is key to implementing friction compensation. However, traditional genetic identification algorithms have the shortcomings of a premature solution, slow convergence, and poor accuracy. To address these shortcomings, this paper proposes an improved adaptive genetic identification algorithm. The proposed algorithm selects evolutionary processes adaptively according to the population concentration in the initial stage of population evolution. Moreover, it adjusts the crossover probability and the mutation probability to identify a local optimum accurately and converge to the global optimum rapidly. During the late stage of population evolution, the accuracy of the global optimal solution can be improved by reducing the search range of identification parameters. The simulation results show that the relative error of the model parameter values identified by the proposed algorithm is reduced to less than 1% and the convergence speed is faster. Compared with the existing traditional genetic algorithm and adaptive genetic algorithm, the overall performance of the proposed method is better. This study provides a feasible and highly accurate identification method for parameter identification of friction models used in electro-hydraulic servo systems.

## 1. Introduction

The electro-hydraulic servo system has the advantages of high-power density, fast response speed, and high control precision, and is widely used in aviation, aerospace, and ships [1,2,3]. There are many nonlinear factors when the hydraulic cylinder of an electro-hydraulic servo system is moving at a low speed, and friction is an important inherent nonlinear disturbance factor affecting the dynamic performance of electro-hydraulic servo systems which causes low-speed crawling, jitter, and decreased tracking accuracy [4,5,6]. Therefore, it is of great significance to study the nonlinear friction effect in electro-hydraulic servo systems and put forward the corresponding methods to reduce the influence of friction effect on system stability and accuracy as much as possible.

In terms of reducing the impact of friction, it is more practical and cheaper to compensate nonlinear friction than pure mechanical methods, such as improving the contact surface material and lubrication conditions [7]. For compensating nonlinear friction, the key link is to establish an accurate friction model and accurately determine the parameters of this model, which is helpful in carrying out friction compensation of precision mechanical system engineering application research, such as trace tracking and dynamic prediction. So far, many effective mathematical models that describe friction characteristics have been proposed, one after another, such as the Coulomb model, Stribeck model, Dahl model, Leuven model, LuGre model, etc. Among them, the LuGre friction model is a relatively perfect friction model, which can accurately describe the characteristics of viscous slip motion, friction hysteresis, pre-sliding displacement, and variable maximum static friction force in the friction process [8]. Therefore, researchers generally use the LuGre friction model to accurately describe the static and dynamic characteristics of friction at present, and propose the corresponding friction compensation measures, such as adaptive compensation [9], active disturbance rejection compensation [10], and friction observer compensation [11]. Freidovich et al. ignored the influence of external disturbance and designed a feedback controller based on the LuGre model by designing a high-gain state observer to eliminate or reduce the influence of external interference so that the nonlinear friction compensation is completed [12].Wang et al. designed the corresponding nonlinear friction observer based on the LuGre friction model and verified its performance [13]. Jiang et al. established a mathematical model of a digital hydraulic cylinder based on the LuGre friction model and proved that the addition of adaptive friction compensation control can effectively reduce system static error, suppress system limit loop oscillation, and improve the overall performance of the digital hydraulic cylinder [14]. Zhang et al., based on the LuGre dynamic friction model, proposed a nonlinear controller based on active disturbance rejection control to compensate the nonlinear effect of friction, while the simulation results verified the feasibility and the effectiveness of their method [15].

The LuGre friction model is composed of a set of nonlinear equations, including six parameters, namely, two dynamic parameters which are the bristle stiffness coefficient and damping coefficient, and four static parameters which are coulomb friction, static friction, viscous friction coefficient, and Stribeck velocity. These parameters are based on the experimental data of observable state variables and calculated by the identification algorithm. The acquisition of model parameters is the premise of the implementation of these friction compensation measures, and the more accurate the parameters are, the more effective the compensation is. However, since the coupling between the six different parameters as well as the internal state variables involved in the model are difficult to measure in the actual system, it is very difficult to determine the parameters of the LuGre friction model, especially the two dynamic parameters. In addition, due to the large number of parameters to be identified, the calculation cost also increases significantly, which also brings great difficulties to the parameter identification of the LuGre friction model. For this reason, many scholars have carried out relevant research. Xu studied a new identification method for determining the parameters of dynamic systems from step responses based on the Newton algorithm. The simulation results displayed that the obtained models can effectively capture the dynamics of the systems [16]. Canudas et al. proposed a two-step identification method based on local linearization. The four static parameters and two dynamic parameters in the model were identified in two steps using the least square method [17]. Based on the two-step method, Liu et al. identified six parameters using the least square method and the boundary error estimation method based on interval analysis, respectively, avoiding the defects of traditional identification methods such as the difficulty in determining the initial value [8]. Nevertheless, the traditional identification algorithms have some limitations. For example, the Newton identification algorithm is sensitive to the initial value selection and the result is easy to diverge; the two-step identification method is singular and the calculation amount is large.

In addition to the above methods, among the current model identification algorithms, the intelligent algorithms represented by the simulated annealing algorithm (SAA) [18], the neural network algorithm (NNA) [19], particle swarm optimization (PSO) [20], and the genetic algorithm (GA) [21], with their excellent solution set generation and search ability, have gradually replaced the traditional classical algorithms such as Newton iteration and the least square method. These methods are more and more widely used, especially for multi-parameter and multi-extremum combination optimization and system identification problems. For the application of these methods in the field of friction model parameter identification, Irakoze et al. used a PSO algorithm to identify the parameters of the LuGre friction model and verified the effectiveness of the algorithm through experiments [22]. Chen et al. used the Coulomb model to establish the friction model of a projectile propulsion system, and identified the parameters of the friction model based on the simulated annealing algorithm. The identification results showed that the algorithm had good identification accuracy, but its convergence speed was slow [23]. In fact, these intelligent algorithms also have some defects, for example, the generalization performance of NNA is poor when processing a large amount of data [24], the convergence speed of SAA is slow, and the PSO algorithm has the problem of low precision and easy divergence [20]. Considering the multi-parameter characteristics of the friction model established by the research object, this paper intends to use the GA with relatively ideal parallel iteration ability as the identification method of the friction model.

Although the genetic identification algorithm is not sensitive to initial value selection and has global search ability, the traditional genetic identification algorithm adopts fixed control parameters, resulting in it possessing a poor global search ability and easily causing a ‘premature’ phenomenon to occur, falling into local optimum [25,26]. In order to solve the problems existing in the traditional genetic identification algorithm, many scholars have proposed some improved genetic algorithms such as an improved genetic algorithm, adaptive genetic algorithm, fuzzy genetic algorithm, and so on. These methods improve the defects existing in the traditional genetic algorithm to a certain extent, but there is still room for improvement in terms of convergence speed and accuracy. In this paper, combined with the global search ability of the traditional genetic identification algorithm, a new improved genetic algorithm which is called the adaptive genetic identification algorithm is proposed. By designing the adaptive evolution module and the optimal solution accuracy optimization module, the improved adaptive genetic identification algorithm solves the problems that the traditional genetic identification algorithm causes a ‘premature’ phenomenon, and has slow convergence speed and low solution accuracy. The specific process is: in the early stage of population evolution, according to the concentration of individual population, adaptive adjustment crossover probability, and mutation probability, improve the convergence speed, while avoiding the ‘premature’ algorithm and falling into local optimum; in the later stage of evolution, the search range of identification parameters is reduced adaptively to improve the identification accuracy of the friction model. Based on the improved adaptive genetic identification algorithm, this paper provides a feasible and highly accurate identification method for parameter identification of the friction model of electro-hydraulic servo systems.

Based on the above, a new approach for the parameter identification of the LuGre friction model based on the improved adaptive genetic recognition algorithm is proposed. The main contributions of this paper can be epitomized as follows:

1. On the basis of the traditional genetic algorithm, combining it with the adaptive evolution module and the optimal solution accuracy optimization module, a new parameter recognition algorithm is proposed.

2. Based on the simulation experiments, it is verified that the proposed algorithm can identify the six parameters of the LuGre friction model effectively. In order to verify the advantages of the proposed method, it is compared with the existing methods. The results show that the proposed method can significantly improve the accuracy of parameter identification on the basis of guaranteeing fast convergence speed.

The rest of this paper is arranged as follows: Section 2 primarily demonstrates the detailed description of the LuGre friction model and the identification model; Section 3 displays the theoretical model of the adaptive improved genetic algorithm proposed in this paper in detail; Section 4 uses a simulation example to verify the performance of the proposed parameter identification algorithm and compare it with the existing methods; In Section 5, we summary this paper and discuss the possible work in the future. 

## 2. System Model

### 2.1. LuGre Friction Model

The LuGre friction model is a synthesis of the Dahl model and the Bristle model, which simulates the contact surface of two objects with countless tiny elastic bristles. The restoring force generated by the elastic deformation of the bristles is regarded as a friction force [27]. This model has been widely used to describe friction characteristics of servo systems [12,28], and it is defined as follows:

(1) $$\{\begin{array}{c} F_{f}=\sigma_{0}z+\sigma_{1}\overset{\cdot }{z}+\sigma_{2}v\\ \overset{\cdot }{z}=v-\frac{\left|v\right|}{g(v)}z\\ g(v)=\frac{F_{c}}{\sigma_{0}}+\frac{F_{s}-F_{c}}{\sigma_{0}}e^{-{\left(\frac{v}{v_{s}}\right)}^{2}} \end{array}$$

where *σ*_0_ is the bristle stiffness coefficient; *σ*_1_ denotes the microscopic damping coefficient; *σ*_2_ represents the viscous friction coefficient; *F_c_* denotes the Coulomb friction force; *F_s_* is the maximum static friction force; *v_s_* is the Stribeck velocity; and *z* denotes the average bristle deformation. Accurate determination of the six parameters *σ*_0_, *σ*_1_, *σ*_2_, *F_c_*, *F_s_*, and *v_s_* is the key to the study of friction compensation using the LuGre friction model.

The average bristle deformation *z* of actual systems is difficult to measure. Therefore, it is regarded as an intermediate parameter in the process of friction parameter identification. After discretization, Equation (1) can be converted to the following form:

(2) $$\{\begin{array}{c} F_{f}(k)=\sigma_{0}z(k)+\sigma_{1}\overset{\cdot }{z}(k)+\sigma_{2}v(k)\\ \begin{array}{l} \overset{\cdot }{z}(k)=v(k)-\frac{\left|v(k)\right|}{g(k)}z(k)\\ z(k)=\sum_{i=0}^{k-1}\overset{\cdot }{z}(i)t_{0} \end{array}\\ g(k)=\frac{F_{c}}{\sigma_{0}}+\frac{F_{s}-F_{c}}{\sigma_{0}}e^{-{\left(\frac{v(k)}{v_{s}}\right)}^{2}} \end{array}$$

where *t*_0_ is the sampling period and *k* is the number of sampling points.

### 2.2. Identification Model

In order to study the dynamic characteristics of the bristle in the LuGre model at the adhesion stage without losing generality, the piston rod, and its connectors with mass *m,* is taken as the research object. The object is placed on a fixed horizontal plane under the action of external load *F_n_*, which drives the hydraulic cylinder to produce displacement *x*. The friction force of the electro-hydraulic servo systems is *F_f_*.

According to Newton’s second law, the dynamic equation of the actuator of an electro-hydraulic servo system is given by:

(3) $$m\overset{\cdot \cdot }{x}=F_{n}-F_{f}$$

The state parameter *x* is defined by $x={\left[\begin{array}{cc} x_{1} & x_{2} \end{array}\right]}^{T}$, $u=F_{n}$, where $x_{1}=\overset{\cdot }{x}=v$ and $x_{2}=z$.

Combining Equations (2) and (3), the discrete state equation of the actuator of an electro-hydraulic servo system is given by:

(4) $$\left\{ \begin{array}{l} \overset{\cdot }{x}(k)=A(k)x(k)+B(k)u(k)\\ y(k)=C(k)x \end{array}\right.$$

where the system matrix *A*(*k*), input matrix *B*(*k*), and output matrix *C*(*k*) are, respectively, expressed as follows:

(5) $$A(k)=\left[\begin{array}{cc} -\frac{1}{m}(\sigma_{1}+\sigma_{2}) & \frac{1}{m}(\sigma_{0}-\frac{\sigma_{0}\left|x_{1}(k)\right|}{F_{c}+(F_{s}-F_{c})e^{-{\left(\frac{x_{{}_{1}}(k)}{v_{s}}\right)}^{2}}})\\ 1 & -\frac{\sigma_{0}\left|x_{1}(k)\right|}{F_{c}+(F_{s}-F_{c})e^{-{\left(\frac{x_{{}_{1}}(k)}{v_{s}}\right)}^{2}}} \end{array}\right]$$

(6) $$B(k)=\left[\begin{array}{c} \frac{1}{m}\\ 0 \end{array}\right]$$

(7) $$C(k)=\left[\begin{array}{cc} 1 & 0 \end{array}\right]$$

The objective function in the optimization process is defined as follows:

(8) $$J=\frac{1}{2}{\sum_{i=0}^{k}\left(v(k)-\overset{\wedge }{v}(k)\right)}^{2}$$

where $\overset{\wedge }{v}(k)$ is the velocity identification value of the hydraulic cylinder, and it is calculated by substituting the values of velocity *v*(*k*) and driving force *F_n_*(*k*) of the observable measured hydraulic cylinder into Equations (2)–(7).

The fitness function is defined by:

(9) $$F_{\mathrm{fitness}}=\frac{1}{J}$$

Equations (2)–(9) constitute the parameter identification model of the LuGre friction model of an electro-hydraulic servo system. The collected observable data of velocity *v*(*k*) and driving force *F_n_*(*k*) (*k* = 0, 1, …, *n*) are substituted into the parameter identification model. An appropriate identification algorithm is used to calculate the friction parameters of LuGre friction model.

## 3. Improved Adaptive Genetic Identification Algorithm

The GA is a global search algorithm that simulates the law of biological evolution. It selects individuals that can adapt to a defined environment for replication, crossover, and mutation according to the principle of survival of the strongest individuals to produce a new generation of individuals that are more adaptable to the environment. This process is repeated until the result finally converges to a single individual that is the most adaptable to the environment among all individuals, and this solution represents the global optimal solution to the considered problem.

**Figure 1:**
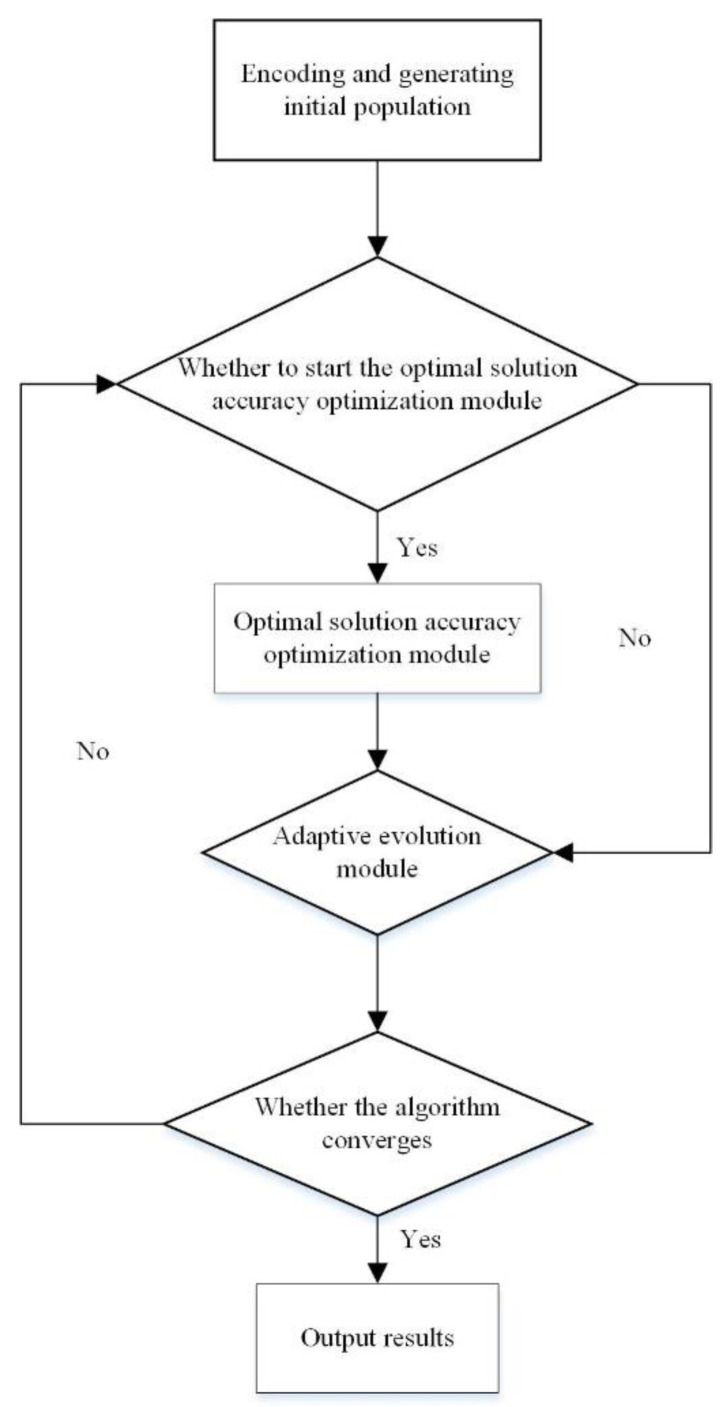
The flowchart of the proposed improved adaptive genetic identification algorithm.

However, the traditional genetic identification algorithm adopts fixed control parameters, resulting in it possessing a poor global search ability and easily causing a ‘premature’ phenomenon to occur, falling into local optimum. To solve these problems, based on the traditional genetic algorithm, this section introduces an improved adaptive genetic identification algorithm consisting of two modules, the adaptive evolution module and the optimal solution accuracy optimization module. The proposed algorithm aims to solve many problems in the traditional genetic identification algorithms, such as premature solutions, slow convergence speed, and poor accuracy. The flowchart of the proposed algorithm is shown in Figure 1.

### 3.1. Adaptive Evolution Module Design

The main function of the adaptive evolution module is to avoid premature solutions and accelerate convergence. Its specific calculation steps are as follows. When the population concentration is higher than or equal to 0.5, differences within the population are small, so the crossover is not effective in generating better individuals. Moreover, much computing time is consumed to execute the algorithm. Therefore, mutation should be first conducted to increase the mutation probability *P*_m_ to make the algorithm able to avoid local optimums and obtain the global optimum. Then, the crossover is performed to reduce the crossover probability *P*_c_. When the population concentration is lower than 0.5, differences within the population are large. Crossover can increase the crossover probability *P*_c_ to ensure the full evolution of individuals to generate better individuals. The follow-up mutation can reduce the mutation probability *P*_m_, i.e., the probability of destroying high-quality individuals.

The population concentration is defined as follows:

(10) $$f_{\mathrm{cratio}}=\frac{\sum_{i=1}^{n}f_{i}/n}{\max(f_{i})}$$

where *f_i_* is the fitness value of individuals in the population.

The crossover probability is calculated by:

(11) $$P_{c}=k_{1}(1-\frac{2\arcsin f_{\mathrm{cratio}}}{\pi })$$

where *k*_1_ is the crossover coefficient.

The mutation probability is calculated by:

(12) $$P_{m}=\frac{2k_{2}\arcsin f_{\mathrm{cratio}}}{\pi }$$

where *k*_2_ is the mutation coefficient.

### 3.2. Optimal Solution Accuracy Optimization Module Design

When the solution obtained by the adaptive evolution module converges to the vicinity of an optimal solution, the optimal solution accuracy optimization module starts. The starting conditions are defined as follows:

(13) $$\left|\max f_{j,i}-\max f_{j-1,i}\right|<\delta \;\mathrm{and}\;k_{0}\geq \alpha m$$

$$(j=k_{0},k_{0}-1,\ldots ,k_{0}-\gamma m+1;\;i=1,2,\ldots ,n)$$

where *f_j,i_* is the fitness value of the *i*th individual in the *j*th evolution; *k*_0_ is the number of evolutions of the population; *m* denotes the total number of evolutions; *n* is the total number of individuals in the population; *δ*, *γ*, and *α* are the starting coefficients of the optimal solution accuracy optimization module.

The optimal solution accuracy optimization module is based on the idea of zoom [29]. The calculation process is as follows. If genes at the highest positions of *βn* (0 < *β* < *α*) individuals with the highest fitness values in the population are the same, the genes at the highest positions of these *βn* individuals are released (emptied) to store in a decoding formula, and the genes of the rest positions are shifted to the left by one position, i.e., the *q-*th position is changed to (*q* + 1)th position, where *q* = 1, 2, …, (*l*− 1), and *l* denotes the individual length. The emptied-first (lowest) position is used to contain a new gene 0. Then, randomly generated (1−β)n individuals are used to replace the remaining unchanged (1−β)n individuals. In this way, the obtained *n* individuals constitute a new initial population. The adaptive evolution module is then used to calculate and generate optimal individuals in this generation.

After *r* iterations of accuracy optimization of the optimal solution for the population, the decoding formula of the individuals is used as follows with coding $h_{l}h_{l-1}\ldots h_{1}$:

(14) $$d_{\left(h_{l}h_{l-1}\ldots h_{1}\right)}=a+\sum_{i=1}^{r}\frac{u_{i}}{2^{i-1}}\frac{2^{l-1}}{2^{l}-1}(b-a)+\sum_{i=1}^{l}h_{i}{}^{\left(r\right)}2^{i-1}\frac{b-a}{2^{r}(2^{l}-1)}$$

where *a* is the lower limit of the encoded parameter, *b* denotes the upper limit of the encoded parameter, and *u_i_* the gene position released at the *i*th time.

Equation (14) shows that the optimal solution accuracy optimization module can narrow the parameter search range from (*b* − *a*) to $(b-a)/2^{r}$, thus improving the search resolution and solution accuracy under the condition of unchanged length of individual coding.

## 4. Simulation Results

For the improved adaptive genetic recognition algorithm, binary coding and a roulette wheel selection method were adopted to achieve replication and single-point crossover with MATLAB programming. The parameter settings of the proposed algorithm are provided in Table 1.

A dynamic simulation model of an electro-hydraulic servo system was constructed using the AMESim software to simulate collected discrete data of hydraulic cylinder velocity *v*(*k*) and hydraulic cylinder driving force *F_n_*(*k*). The parameter settings of the LuGre friction model are provided in Table 2.

**Figure 2:**
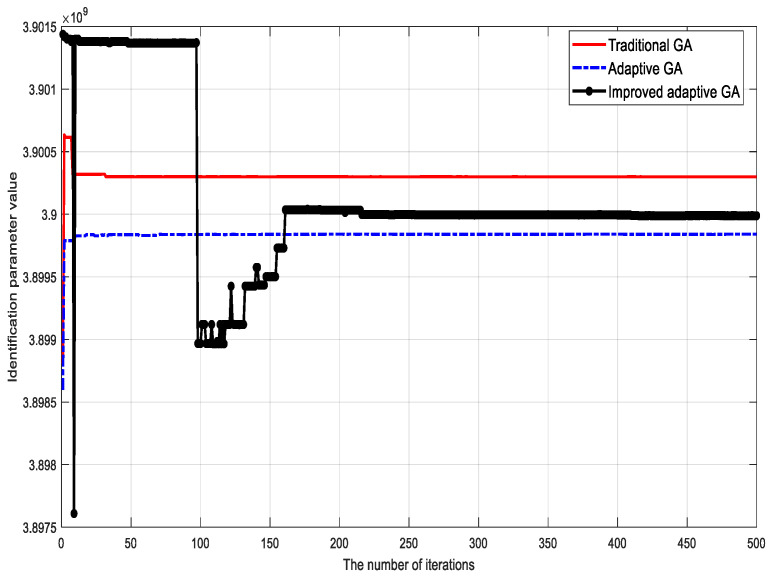
Identification results of bristle stiffness coefficient $\sigma_{0}$.

**Figure 3:**
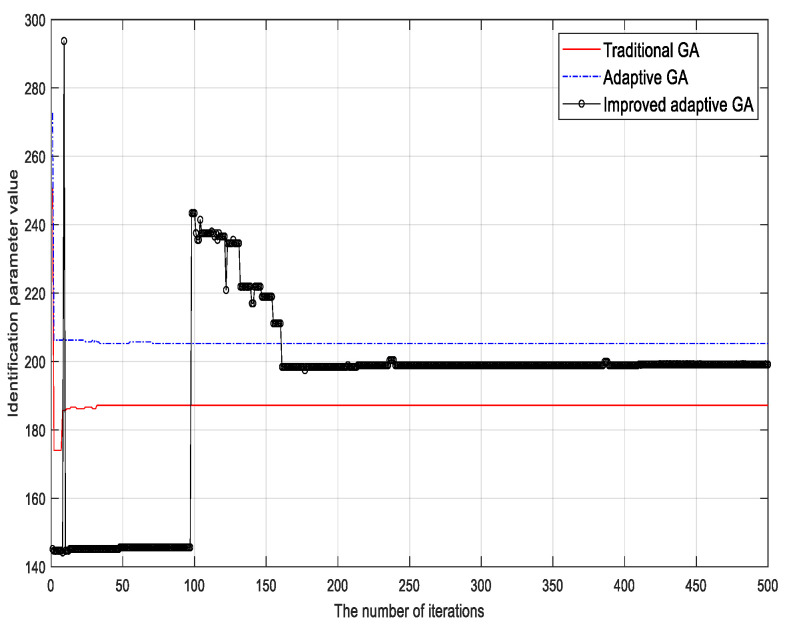
Identification results of micro damping coefficient $\sigma_{1}$.

**Figure 4:**
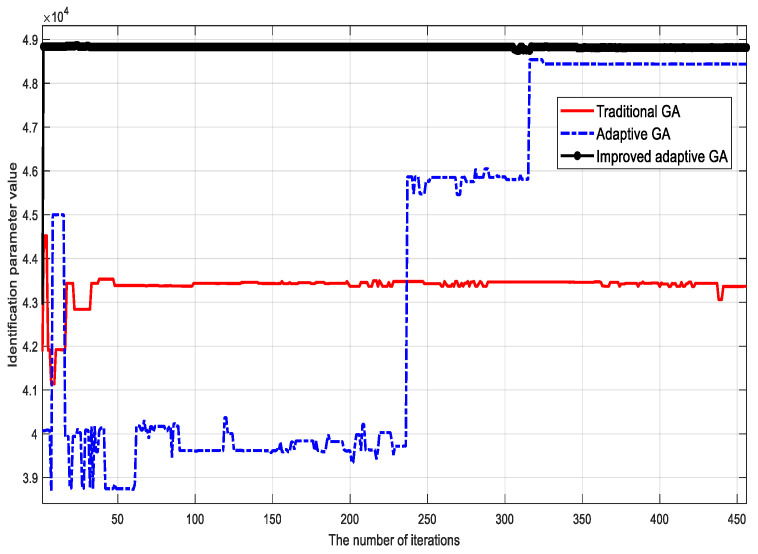
Identification results of viscous friction coefficient $\sigma_{2}$.

**Figure 5:**
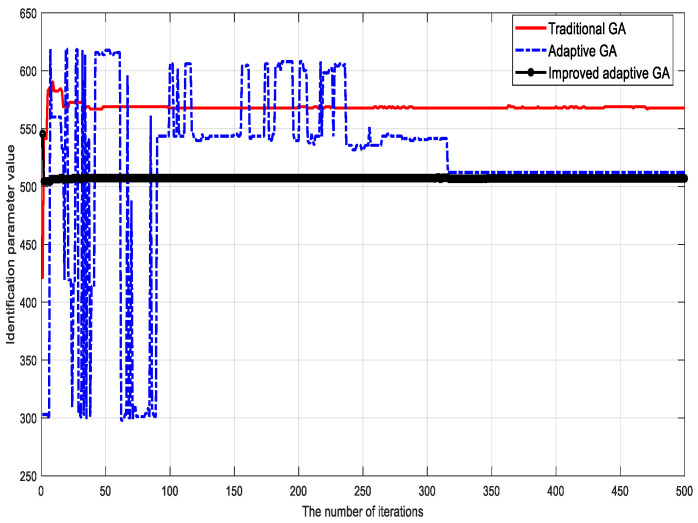
Identification results of coulomb friction $F_{c}$.

**Figure 6:**
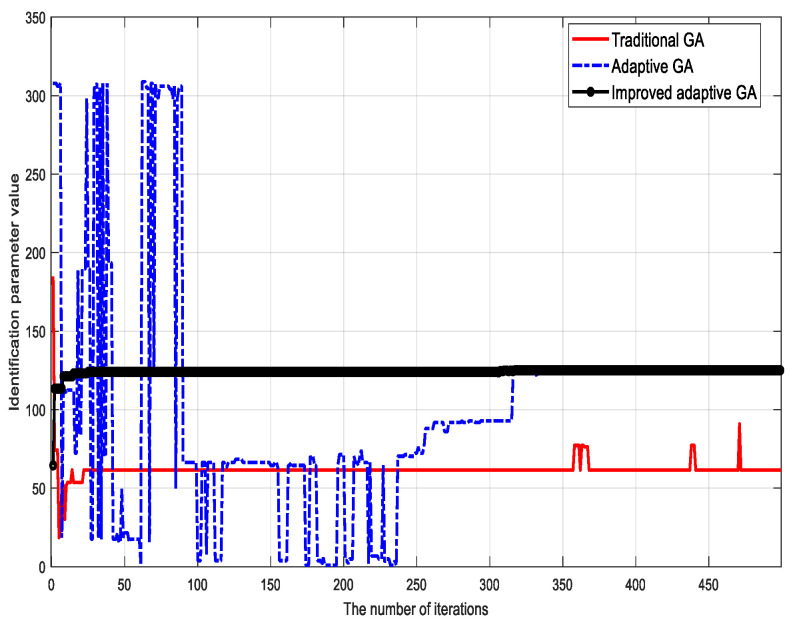
Identification results of difference between maximum static friction $F_{s}$ and coulomb friction $F_{c}$.

**Figure 7:**
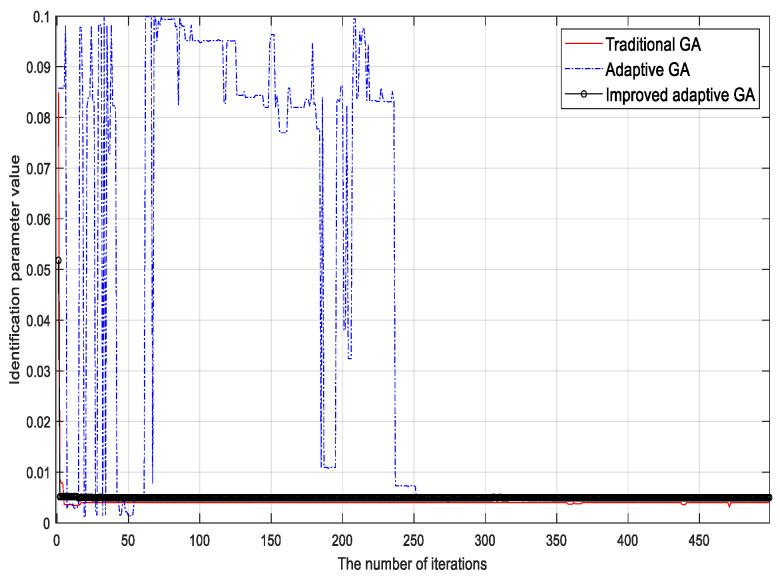
Identification results of Stribeck speed $v_{s}$.

To verify the validity of the proposed algorithm, the traditional genetic identification algorithm, the adaptive genetic identification algorithm, and the proposed (improved) genetic identification algorithm were used to identify the parameters of the LuGre friction model. The parameter identification results corresponding to different methods are shown in Figure 2, Figure 3, Figure 4, Figure 5, Figure 6 and Figure 7.

Based on the identification results, the following conclusions can be drawn:

(1) Although the traditional genetic identification algorithm converges faster, it causes a ‘premature’ phenomenon and falls into a local optimum, causing inaccurate parameter identification results of the friction model and large errors compared with the actual (true) values;

(2) Compared with the traditional genetic identification algorithm, the adaptive genetic identification algorithm effectively alleviates the ‘premature’ phenomenon, but the convergence speed is significantly slow. In addition, the identification results are very unstable in the iterative process;

(3) Compared with the two existing methods above, the improved adaptive genetic algorithm proposed in this paper not only avoids the ‘premature’ phenomenon of the traditional genetic identification algorithm, so as to have more accurate identification results, but also enjoys faster convergence speed than the adaptive genetic identification algorithm. Therefore, the recognition performance of the proposed algorithm is better.

The identification accuracies of the three different algorithms are compared in Table 3. It can be clearly seen that for the six key parameters, the accuracy of the proposed algorithm is significantly improved compared with the two existing methods.

**Figure 8:**
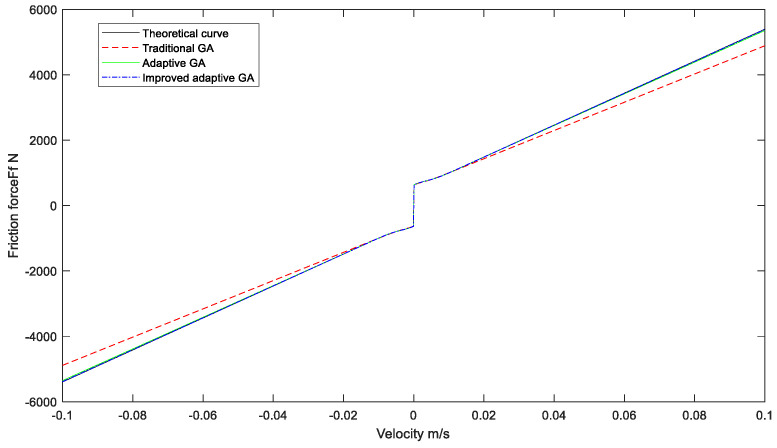
The relationship curve between velocity and friction corresponds to different algorithms.

**Figure 9:**
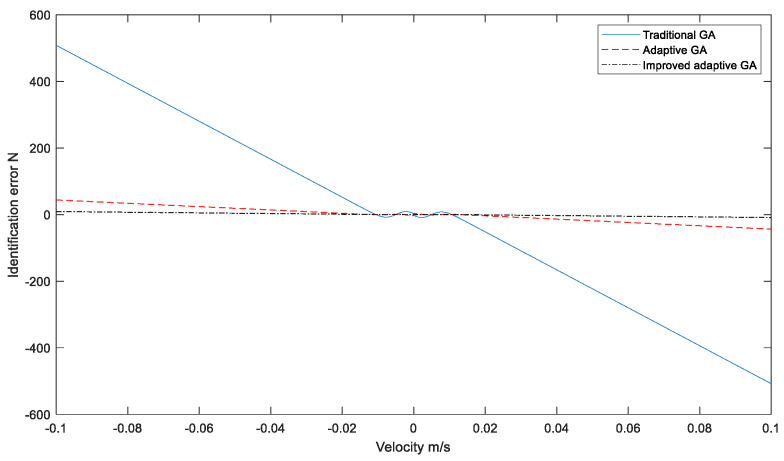
The identification error corresponds to different algorithms.

Based on the optimal parameters obtained by different optimization algorithms, the relation curve between hydraulic cylinder speed and friction force is obtained, as shown in Figure 8 and Figure 9.

## 5. Conclusions

In order to accurately and quickly realize the effective identification of the LuGre friction model parameters, based on the adaptive evolution module design and the optimal solution accuracy optimization module design, this article proposes an improved adaptive genetic identification algorithm to address the shortcomings of the existing parameter identification algorithms. Compared to the traditional genetic algorithms and adaptive genetic algorithms, the proposed identification algorithm can effectively avoid premature solutions falling into a local optimum and improve identification accuracy significantly. The simulation results verify that the proposed algorithm is effective and feasible and has an identification error of less than 1%. Based on the LuGre friction model and the improved adaptive genetic identification algorithm, the research results of this paper are expected to contribute to reducing the impact of nonlinear friction on the electro-hydraulic servo system. However, due to the influence of various factors, the performance of the proposed method is only preliminarily verified in the simulation model. In future research, our work will mainly focus on the following aspects:

(1) We will try to apply the algorithm proposed in this paper to the actual electro-hydraulic servo system so as to verify its performance in practical engineering applications, and make further adaptive improvements to the algorithm for possible problems.

(2) Although the convergence speed of the algorithm proposed in this paper is faster than the adaptive genetic algorithm, it is still significantly slower than the traditional genetic algorithm. The further optimization of the proposed algorithm so as to improve the convergence speed and make the algorithm more suitable for engineering applications is also the focus of future research.

## Figures and Tables

**Table 1:** The parameter settings of the proposed algorithm.

Parameter Type	Value
Search range of the bristle stiffness coefficient *σ*_0_	[0, 5 × 10^9^]
Search range of the microscopic damping coefficient *σ*_1_	[0, 1000]
Search range of the viscous friction coefficient *σ*_2_	[0, 50,000]
Search range of the Coulomb friction force *F_c_*	[0, 1000]
Search range of the maximum static friction force *F_s_*	[0, 1000]
Search range of the Stribeck velocity *v_s_*	[0.001, 0.1]
Sampling period *t*_0_	0.001 s
Crossover coefficient *k*_1_	0.8
Mutation coefficient *k*_2_	0.7
Starting coefficient *γ*	0.1
Starting coefficient *α*	0.6
Constant *β*	0.4
Number of iterations *m*	500

**Table 2:** The parameter settings of the LuGre friction model.

Parameter Type	Value
Bristle stiffness coefficient σ0	3.9 × 109 N/m
Microscopic damping coefficient σ1	200 N/(m/s)
Viscous friction coefficient σ2	48,900 N/(m/s)
Coulomb friction force Fc	506 N
Maximum static friction force Fs	632 N
Stribeck velocity vs	0.005 m/s
Piston rod mass M	200 kg

**Table 3:** Identification accuracies of the three algorithms.

Parameter Type	Different Genetic Algorithm	Identification Value	Error (%)
Bristle stiffness coefficient *σ*_0_	Traditional	3.9003 × 10^9^	0.0077
	Adaptive	3.8996 × 10^9^	0.010
	Improved adaptive	3.8999 × 10^9^	0.0026
Microscopic damping coefficient *σ*_1_	Same as above	187.19	6.4
		205.23	2.6
		201.37	0.69
Viscous friction coefficient *σ*_2_		4.32 × 10^4^	57
		4.84 × 10^4^	1.0
		4.88 × 10^4^	0.20
Coulomb friction force *F_c_*		567.94	12
		512.22	1.2
		507.01	0.20
Difference between the maximum static friction force *F_s_* and the Coulomb friction force *F_c_*		61.58	51
		123.20	2.2
		125.10	0.71
Stribeck velocity *v_s_*		0.00400	20
		0.00480	4.0
		0.00498	0.4

## Data Availability

Not applicable.
